# Development and Validation of Predictive Risk Scores for Ovarian Clear Cell Carcinoma: A Penalized Regression Model

**DOI:** 10.1002/cam4.71118

**Published:** 2025-08-07

**Authors:** Shohei Iyoshi, Ryo Emoto, Nobuhisa Yoshikawa, Kosuke Yoshida, Masato Yoshihara, Satoshi Tamauchi, Akira Yokoi, Yusuke Shimizu, Yoshiki Ikeda, Kaoru Niimi, Michiyasu Kawai, Shigeyuki Matsui, Hiroaki Kajiyama

**Affiliations:** ^1^ Department of Obstetrics and Gynecology Nagoya University Graduate School of Medicine Nagoya Japan; ^2^ Institute for Advanced Research, Nagoya University Nagoya Japan; ^3^ Department of Biostatistics Nagoya University Graduate School of Medicine Nagoya Japan; ^4^ Department of Obstetrics and Gynaecology Toyohashi Municipal Hospital Toyohashi Japan

**Keywords:** clear cell carcinoma, machine learning, ovarian cancer, penalized regression, prediction model

## Abstract

**Background:**

The precision management of ovarian clear cell carcinoma (OCCC) faces limitations due to the absence of personalized prognostic tools. This study aimed to establish predictive risk scores to enable effective stratification and tailored treatment of OCCC.

**Methods:**

Retrospective data from 206 OCCC patients treated between 2004 and 2019 at two hospitals were analyzed. Penalized regression models were utilized to develop three risk scores based on preoperative laboratory results and intraoperative findings. These scores underwent internal and external validation.

**Results:**

The median follow‐up periods were 65.7 and 44.0 months for the derivation and validation cohorts, respectively. In internal validation with the derivation cohort, all three risk scores effectively identified the high‐risk group for tumor recurrence. Upon validation with the external cohort, Risk Score 3, which incorporated variables selected in most cross‐validations by the penalized regression (Elastic Net), distinctly differentiated the high‐ and low‐risk groups (*p* = 0.03). Risk Score 2, consisting solely of preoperatively available variables, also demonstrated marginal significance (*p* = 0.08).

**Conclusion:**

Our findings underscore the significance and utility of the developed risk scores in tailoring personalized treatments for patients with OCCC.

## Graphical abstract

Preoperative blood test results and information obtained during surgery were used for establishing risk scores to predict high‐risk groups of ovarian clear cell carcinoma recurrence. Variable selection using machine‐learning algorithms showed higher predictive ability than that based on clinicians' experience and insights.

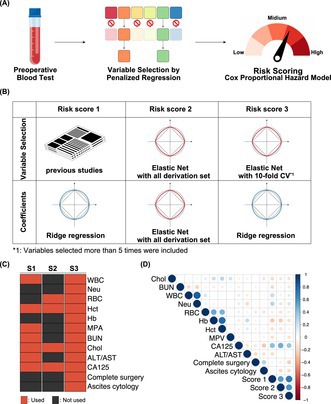

## 1 Introduction

Ovarian clear cell carcinoma (OCCC) presents a distinctive challenge within the realm of ovarian cancer (OvCa) due to its unique clinical and molecular characteristics [1, 2, 3]. While OCCC constitutes approximately 25% of OvCa cases in East Asia, its prevalence is less than 10% in Europe and North America [4, 5]. The importance of OCCC is emphasized by its resistance to conventional platinum‐based chemotherapy and its association with poorer outcomes, particularly in advanced stages, which are even less favorable than those of high‐grade serous ovarian cancer (HGSOC), the most prevalent subtype with poor prognosis. Furthermore, previous research has highlighted the presence of occult metastasis in early‐stage OCCC, and the chemoresistant nature of recurrent OCCC tumors [6]. The prognostic heterogeneity of early‐stage OCCC cases has also been emphasized [7, 8, 9]. Therefore, prognostic biomarkers for predicting OCCC recurrence hold significant clinical importance in achieving precise and tailored treatment for patients with OCCC.

Unlike other histotypes of OvCa, OCCC typically manifests at an earlier stage in younger women [10]. However, despite this seemingly favorable diagnostic scenario, the prognosis for OCCC patients remains challenging compared to those with HGSOC [11]. This paradox is partly attributed to the distinct molecular pathogenesis of OCCC. While HGSOC is often characterized by *TP53* mutations, OCCC frequently displays mutations in *ARID1A* [5, 12, 13]. These significant molecular distinctions are pivotal for comprehending the unique behavior of OCCC and highlight the need for specialized prognostic tools tailored to these specific molecular characteristics.

Traditionally, prognostication for OCCC prognosis has relied on clinicopathological factors and biomarkers such as CA19‐9 and HE4, in addition to clinical stage [14]. Recently, Wu et al. demonstrated that tumor size could serve as an independent prognostic factor for stage I OCCC in a large patient cohort [15]. Our research group has also reported on the prognostic significance of the neutrophil‐to‐lymphocyte ratio or nutritional index in early‐stage OCCC [16, 17]. Although these factors provide some prognostic value, they often lack precision in delivering personalized risk assessments, largely due to their dependence on clinician experience and judgment. Moreover, many of these studies lack external validation, which limits their broader applicability and generalizability across diverse clinical settings.

Recently, the significance of machine‐learning (ML)/deep‐learning (DL) algorithms in the exploration of prognostic clinical biomarkers and the development of prognostic risk scores has been widely recognized [18], and the usefulness was shown in the OvCa cases as well [19]. Acknowledging the limitations of previous studies concerning OCCC, our study delves into the use of ML algorithms to develop more advanced and accurate prognostic models for OCCC. Through the utilization of penalized regression models, we enhance variable selection and coefficient estimation, thereby improving the predictive accuracy of our risk scores. This methodology signifies a notable progression beyond conventional approaches, providing a more data‐driven and objective framework for prognostic assessment. In contrast to the majority of earlier studies, the generated risk scores underwent further validation with an external cohort obtained from a different hospital.

## 2 Materials and Methods

### 2.1 Study Participants

We retrospectively reviewed a consecutive series of 107 and 99 patients with OCCC who underwent surgical treatment at Nagoya University Hospital and Toyohashi Municipal Hospital, respectively, between 2004 and 2019. Ethical approval for this study was obtained from the Ethics Committee at Nagoya University (No. 2006‐0357), adhering to the principles outlined in the Declaration of Helsinki. Due to the retrospective nature of the study, the need for written consent was waived.

### 2.2 Surgery, Chemotherapy, Follow‐Up, and Laboratory Data

Each patient's treatment regimen, which encompassed both surgical intervention and chemotherapy, was tailored based on individual factors such as age, stage of the disease, and ECOG (Eastern Cooperative Oncology Group) performance status. The primary surgical approach involved a comprehensive procedure known as complete staging laparotomy, which included total hysterectomy, bilateral salpingo‐oophorectomy, infracolic omentectomy, and systemic retroperitoneal lymphadenectomy. However, for elderly patients facing severe complications or young patients desiring fertility preservation, as well as the cases in which tumor burden was too high, conservative surgery was performed, typically involving at least unilateral salpingo‐oophorectomy with peritoneal evaluation. In this study, we named the former group the complete surgery group and the latter group the incomplete surgery group. It was recommended that all patients diagnosed with OCCC undergo adjuvant chemotherapy consisting of 3–6 cycles. This regimen typically combined paclitaxel (175 mg/m^2^, day 1) and carboplatin (area under the curve 5, day 1) every 3–4 weeks. In instances where patients exhibited sensitivity to alcohol, docetaxel was administered instead of paclitaxel. In select cases, some patients received either irinotecan monotherapy or a combination therapy of irinotecan and cisplatin. Additionally, in some recent cases, bevacizumab was introduced as part of the treatment regimen. Postoperative monitoring commenced with monthly assessments during the initial year and continued beyond the second year. Evaluations during this period involved the measurement of tumor markers and regular pelvic examinations using various imaging modalities, including ultrasonography, magnetic resonance imaging, computed tomography, and positron emission tomography. Diagnosis of tumor recurrence was established upon the detection of ascites, a palpable mass, or elevated tumor markers, in accordance with the criteria set forth by the Gynecologic Cancer InterGroup [20]. Treatments for recurrent tumors, such as surgery and chemotherapy, were determined based on independent factors such as the recurrent site and the number of recurrent lesions. Preoperative laboratory data were extracted from clinical records and utilized in this study. In most cases, preoperative blood samples were obtained within 1 month prior to the initial surgery. Several blood test parameters, including total cholesterol (Chol), blood urea nitrogen (BUN), white blood cell (WBC), mean platelet volume (MPV), neutrophil count (Neu), CA125, and ALT/AST, underwent analysis following logarithmic transformation and standardization. Other values, such as red blood cell (RBC), hemoglobin (Hb), and hematocrit (Hct), were standardized before utilization. Missing values for continuous variables were imputed by the mean, and for categorical variables, by the mode.

### 2.3 Prognostic Models for Predicting Progression‐Free Survival (PFS)

The data set from patients treated at Nagoya University Hospital (*n* = 107) constituted the derivation cohort, whereas data from patients treated at Toyohashi Municipal Hospital (*n* = 99) served as the external validation cohort. Multivariate Cox regression analysis was conducted on the derivation cohort to generate prediction scores for estimating PFS. Subsequently, these scores' predictive accuracy was evaluated using the validation cohort.

The process of variable selection for predicting the risk of disease progression involved a dual approach integrating clinical expertise from two seasoned gynecologic oncologists with ML methodologies. This method identified three variable sets for further analysis. Risk Score 1 comprised established prognostic variables obtained from preoperative blood tests. Risk Score 2 was developed through Elastic Net penalized regression applied to the entire derivation data set. Risk Score 3 encompassed variables most frequently selected across subsets of the derivation data set utilizing Elastic Net in tenfold cross‐validation (CV).

For Risk Scores 1 and 3, Cox regression models with ridge penalties were utilized to estimate the coefficients of selected variables, thereby generating a continuous prediction score. Conversely, for Risk Score 2, coefficients obtained from the Elastic Net analysis conducted on the entire derivation cohort data set were directly employed. The K‐means method was applied to determine thresholds for distinguishing high‐ and low‐risk groups from the continuous scores generated by all risk scores.

Nested CV was employed to internally assess the accuracy of the prediction scores. This process involved optimizing the penalization parameter within the inner CV loop and evaluating predictive accuracy in the outer CV loop. In external validation, the prediction accuracy of the proposed scores was evaluated across the entire validation set. Harrell's concordance index (C‐index) was calculated for both internal and external validation to gauge the accuracy of the predicted scores. Survival curves for the high‐ and low‐risk groups were constructed using the Kaplan–Meier method, and differences in time to disease progression between these groups were compared using the log‐rank test. Due to the potential impact of overfitting in internal validation, the log‐rank test was exclusively performed in the external validation phase.

### 2.4 Statistical Analysis

Statistical analyses were carried out using SAS version 9.4 (SAS institute, Cary, NC) and R statistical software version 4.1.1 (R Foundation for Statistical Computing, Vienna, Austria). Survival and glmnet packages were used for Cox, ridge, and Elastic Net regression. Comparisons of continuous variables between groups were conducted using Student's *t*‐test (normal distribution) and the Mann–Whitney *U* test (non‐normal distribution). Categorical variables were analyzed using the chi‐squared test or Fisher's exact test. In prognostic analyses, two parameters were assessed: overall survival (OS) and PFS. OS and PFS were defined as the duration from the initial surgery to death or tumor progression, respectively. The Kaplan–Meier method was employed to compare OS and PFS between groups, with differences in survival evaluated using the log‐rank test.

## 3 Results

### 3.1 Baseline Characteristics of the Patients

A total of 206 patients were analyzed, with median follow‐up periods of 65.7 and 44.0 months for the derivation and validation sets, respectively. During these periods, disease progression occurred in 30 and 29 patients, while 22 and 29 deaths were recorded in the derivation and validation cohorts, respectively. Comparing the derivation and validation data sets, there were no significant differences in mean age or clinical stage distribution (Table 1). However, a notable difference was observed in the ratio of incomplete surgeries, attributed partly to the difference in the roles of a university hospital and an affiliated hospital, indicating institutional discrepancies. These differences also could influence the selection of adjuvant chemotherapy regimens. Nevertheless, no significant differences were identified in other factors, such as the volume of ascites or levels of tumor markers. Overall, despite the noted institutional variations, it was concluded that these data sets are suitable for use as derivation and validation sets.

**TABLE 1:** Baseline characteristics of patients in the derivation and validation sets.

Category	Derivation cohort (*n* = 107)	Validation cohort (*n* = 99)	*p*
Age (mean (SD))	53.85 (9.81)	55.09 (11.52)	0.405
FIGO stage (%)			0.441
I	76 (71.0)	63 (63.6)	
II	8 (7.5)	11 (11.1)	
III	21 (19.6)	20 (20.2)	
IV	2 (1.9)	5 (5.1)	
Incomplete surgery (%)	39 (36.4)	55 (55.6)	0.009
Ascites accumulation (%)	76 (71.0)	71 (71.7)	1
Positive ascites cytology (%)	58 (54.2)	50 (50.5)	0.695
CA125 (median [IQR])	53.25 [22.17, 171.88]	106.20 [29.48, 271.72]	0.059
CA19‐9 (median [IQR])	28.00 [11.00, 64.05]	31.65 [11.97, 96.25]	0.337

*Note:* Data are presented as mean ± SD or proportion (%). Student's *t*‐test, Mann–Whitney *U* test, chi‐squared test, or Fisher's exact test was used as appropriate.

Abbreviations: CA, cancer antigen; SD, standard deviation.

### 3.2 Study Design and Prognostic Model Formulation

**FIGURE 1:**
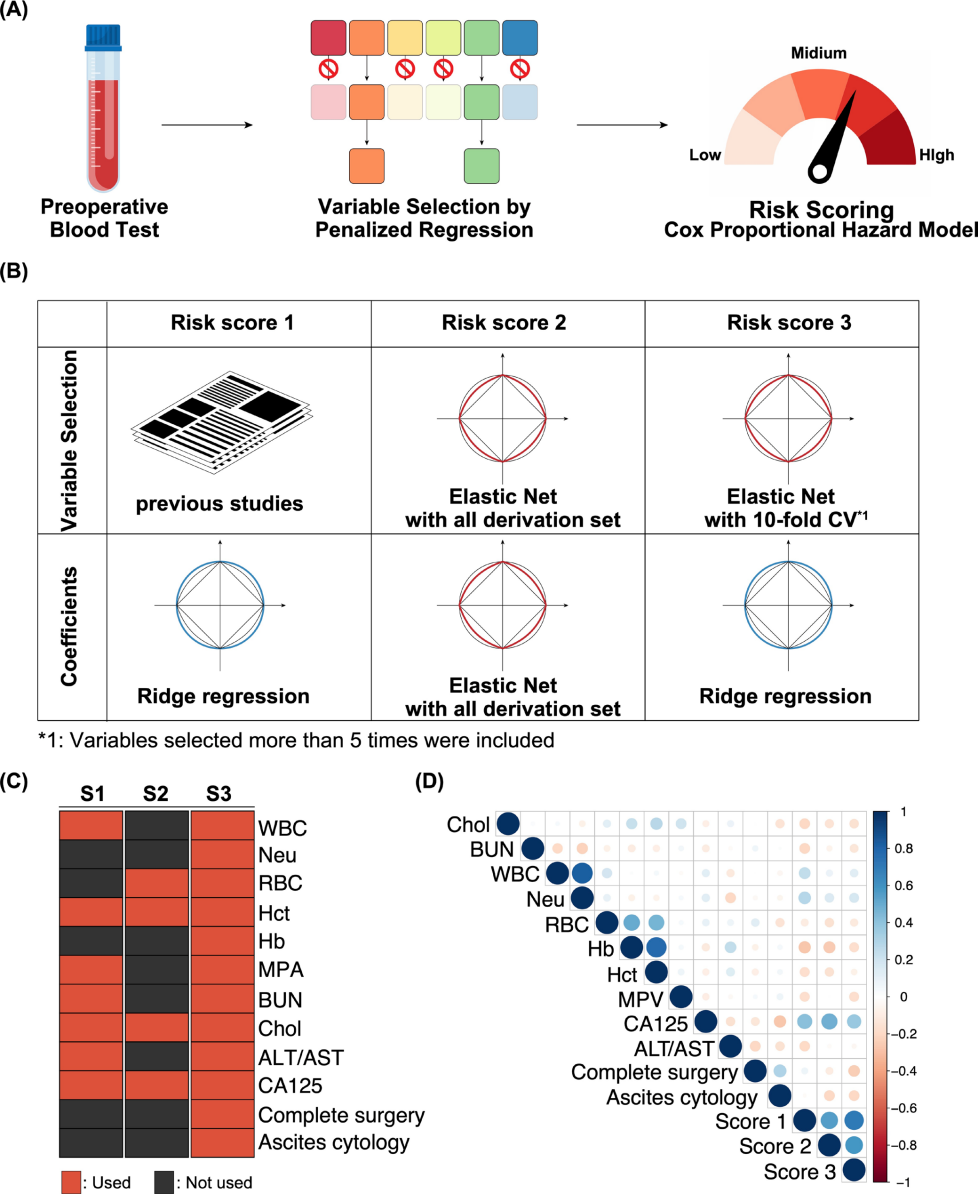
Overview of study design and variable selection. (A) Diagram illustrating the study design. (B) Description of methods employed in variable selection and coefficient estimation for each risk score. (C) List of selected variables for each risk score. (D) Correlation matrix displaying the relationships between variables included in Risk Scores 1–3. Numeric variables such as total cholesterol (Chol), blood urea nitrogen (BUN), white blood cell (WBC), mean platelet volume (MPV), neutrophil count (Neu), CA125, and ALT/AST were used after logarithmic transformation and standardization, whereas red blood cell (RBC), hemoglobin (Hb), and hematocrit (Hct) were used after standardization. Pearson correlations were calculated between numeric variables, polyserial correlations between numeric and ordinal variables, and polychoric correlations between ordinal variables.

The methodological framework of our study is depicted in Figure 1A,B, involving the selection of variables to compute prognostic risk scores based on preoperative blood tests and surgical details. This process adhered to the principles underlying each risk score, employing appropriate coefficient values and calculating each score as the sum of the product of its variables and coefficients (Table 2). For Risk Score 1, we prioritized insights from previous studies, manually selecting variables available from preoperative laboratory test results reported to be associated with the clinical outcome of OvCa. Coefficients for this score were derived from the results of a Cox regression model with ridge penalties applied to the entire derivation cohort data set. In the case of Risk Score 2, emphasis was placed on the direct outcomes of the Elastic Net model. Variables and corresponding coefficients were obtained by analyzing the entire derivation cohort data set using this model. Regarding Risk Score 3, our aim was to optimize the utility of the Elastic Net model's output. We conducted tenfold CV, selecting variables for each CV subset. Ultimately, variables chosen in more than 5 out of 10 CV steps were included in the model (Figure S1). The corresponding coefficients were adapted from the results of ridge regression applied to the entire derivation cohort data set. Thresholds for classifying patients into high‐ and low‐risk groups were determined using the K‐means method for all three risk scores. The selected variables in each risk score are summarized in Figure 1C, with the correlation matrix of these variables displayed in Figure 1D. As expected, correlation scores among the three scoring systems are reasonably high but not excessive, indicating that our intention to compare the three different strategies is feasible. Finally, the comparison of patient characteristics between the high‐ and low‐risk groups is outlined for each risk score in Table S1.

**TABLE 2:** Formulas for calculating each risk score.

	Risk score 1	Risk score 2	Risk score 3
	Coefficients	Coefficients	Coefficients
Incomplete surgery	0 (*β*_1_)	0 (*β*_1_)	0.178 (*β*_1_)
Positive ascites cytology	0 (*β*_2_)	0 (*β*_2_)	0.190 (*β*_2_)
Cho	−0.128 (*β*_3_)	0.049 (*β*_3_)	−0.099 (*β*_3_)
BUN	−0.118 (*β*_4_)	0 (*β*_4_)	−0.103 (*β*_4_)
WBC	0.121 (*β*_5_)	0 (*β*_5_)	0.077 (*β*_5_)
RBC	0 (*β*_6_)	−0.003 (*β*_6_)	−0.106 (β_6_)
Hb	0 (*β*_7_)	0 (*β*_7_)	−0.073 (*β*_7_)
Hct	−0.184 (*β*_8_)	−0.168 (*β*_8_)	−0.102 (*β*_8_)
MPV	−0.124 (*β*_9_)	0 (*β*_9_)	−0.092 (*β*_9_)
Neutrophil	0 (*β*_10_)	0 (*β*_10_)	0.062 (*β*_10_)
CA125	0.176 (*β*_11_)	0.148 (β_11_)	0.121 (*β*_11_)
ALT/AST	−0.100 (*β*_12_)	0 (*β*_12_)	−0.095 (*β*_12_)
Calculation formula	Score = *β*_1_ *X*_1_ + *β*_2_ *X*_2_… + *β*_12_ *X*_12_		
	*β*_*i*_: coeficient of *i*th variable (*i* = 1, …, 12)		
	*X*_*i*_: value of *i*th variable (*i* = 1, …, 12)		
Threshold	−0.0037	0.0101	0.1949

Abbreviations: ALT, alanine transaminase; AST, aspartate transaminase; BUN, blood urea nitrogen; CA, carbohydrate antigen; Cho, total cholesterol; Hb, hemoglobin; Hct, hematocrit; MPV, mean platelet volume; RBC, red blood cell; WBC, white blood cell.

### 3.3 Risk Scores and Their Performance for Predicting Patients' Prognosis

**FIGURE 2:**
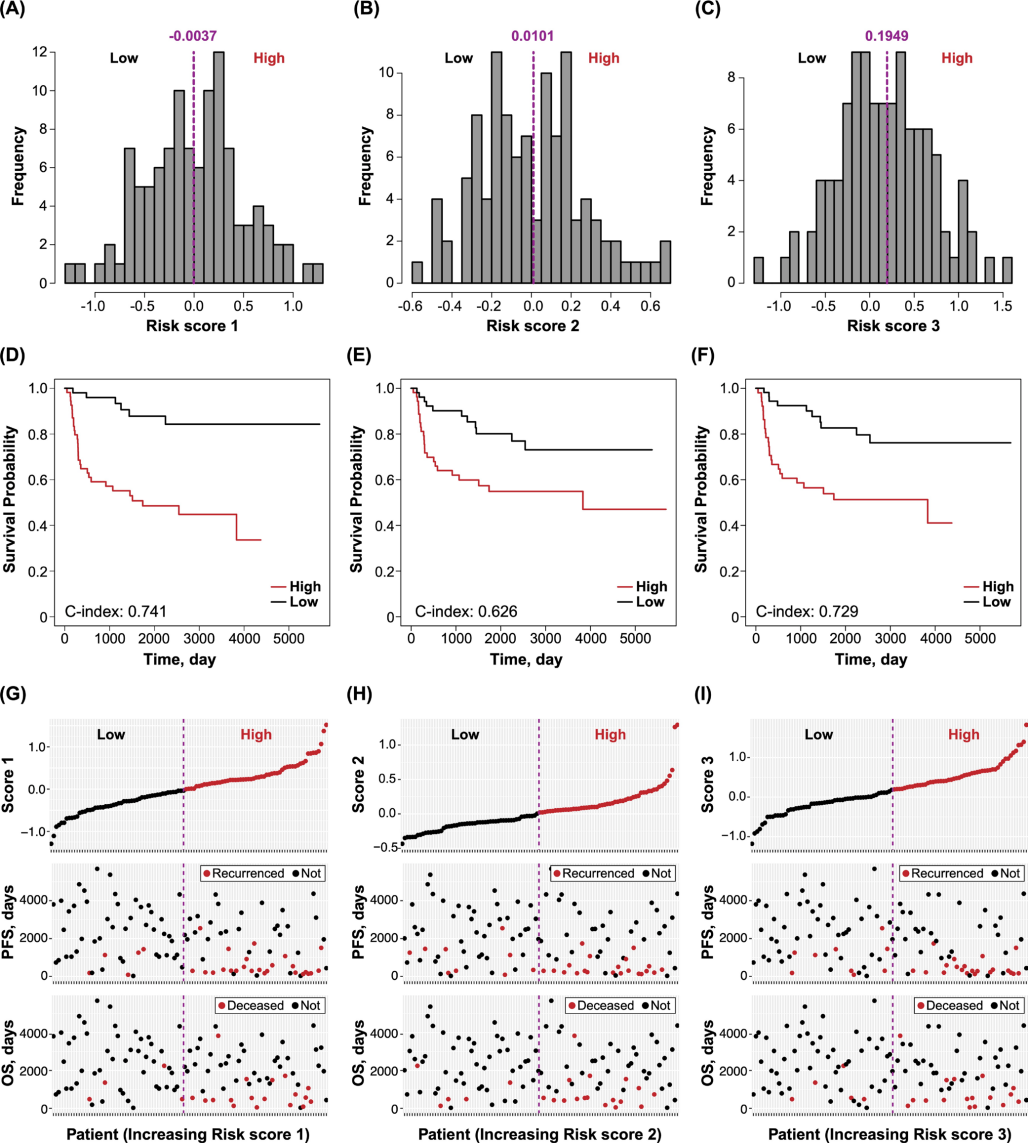
Distribution of each risk score in the derivation set and internal validation. (A–C) Histograms and threshold values depicting Risk Scores 1–3 in the derivation data set. (D–F) Kaplan–Meier curves stratified by high‐ and low‐risk groups based on each risk score. (G–I) PFS and OS plotted against patient ID sorted by each risk score value.

The histograms depicted in Figure 2A–C illustrate the distribution of each risk score within the derivation data set, revealing bimodal‐like distributions indicative of distinct high‐ and low‐risk groups. The thresholds established by the K‐means method effectively separated these two groups. Kaplan–Meier curves, stratified by these two groups, demonstrated that all three risk scores adeptly predicted PFS of patients in the derivation sets, as depicted in Figure 2D–F. Further analysis, plotting PFS and OS against patient IDs sorted by risk score, confirmed that patients with shorter PFS were predominantly classified in the high‐risk group (Figure 2G–I). This observed pattern remained consistent when analyzing OS, particularly noting deceased cases, thereby internally validating the performances of the risk scores.

### 3.4 Risk Scores and Clinical Characteristics of OCCC

**FIGURE 3:**
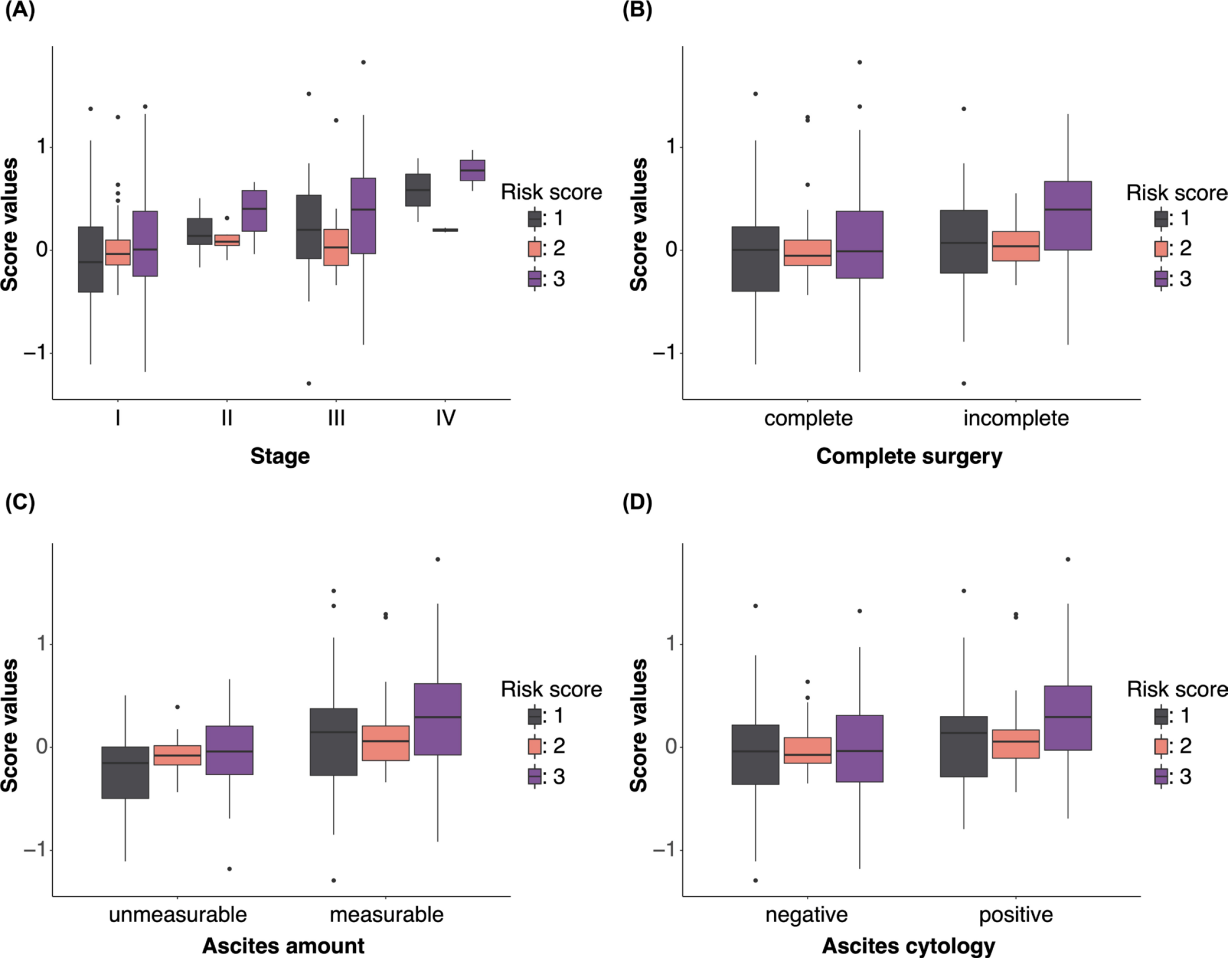
Relationship between risk scores and clinical characteristics of OCCC. (A–D) Box and Whisker plots showing calculated score values in relation to FIGO clinical stages (A), completeness of primary surgery (B), ascites amount (C), and results of ascites cytology (D).

We proceeded to examine the correlation between risk scores and the clinical characteristics of OCCC. Notably, as the disease progressed in stage, there was a tendency for the risk score to increase, a trend clearly depicted in Figure 3A. However, this trend was not as apparent for Risk Score 2, which relies solely on four presurgical blood test factors. Analysis comparing outcomes of complete versus incomplete surgeries revealed higher risk scores for the incomplete surgery cohort, particularly evident for Risk Score 3, as demonstrated in Figure 3B. This finding is attributed to the incorporation of surgery completeness as a variable in the calculation of Risk Score 3. Additionally, the volume of ascites and the results of ascites cytology were associated with the risk scores (Figure 3C,D), with ascites cytology results influencing the calculation of Risk Score 3.

### 3.5 External Validation With a Differently Obtained Data Set

**FIGURE 4:**
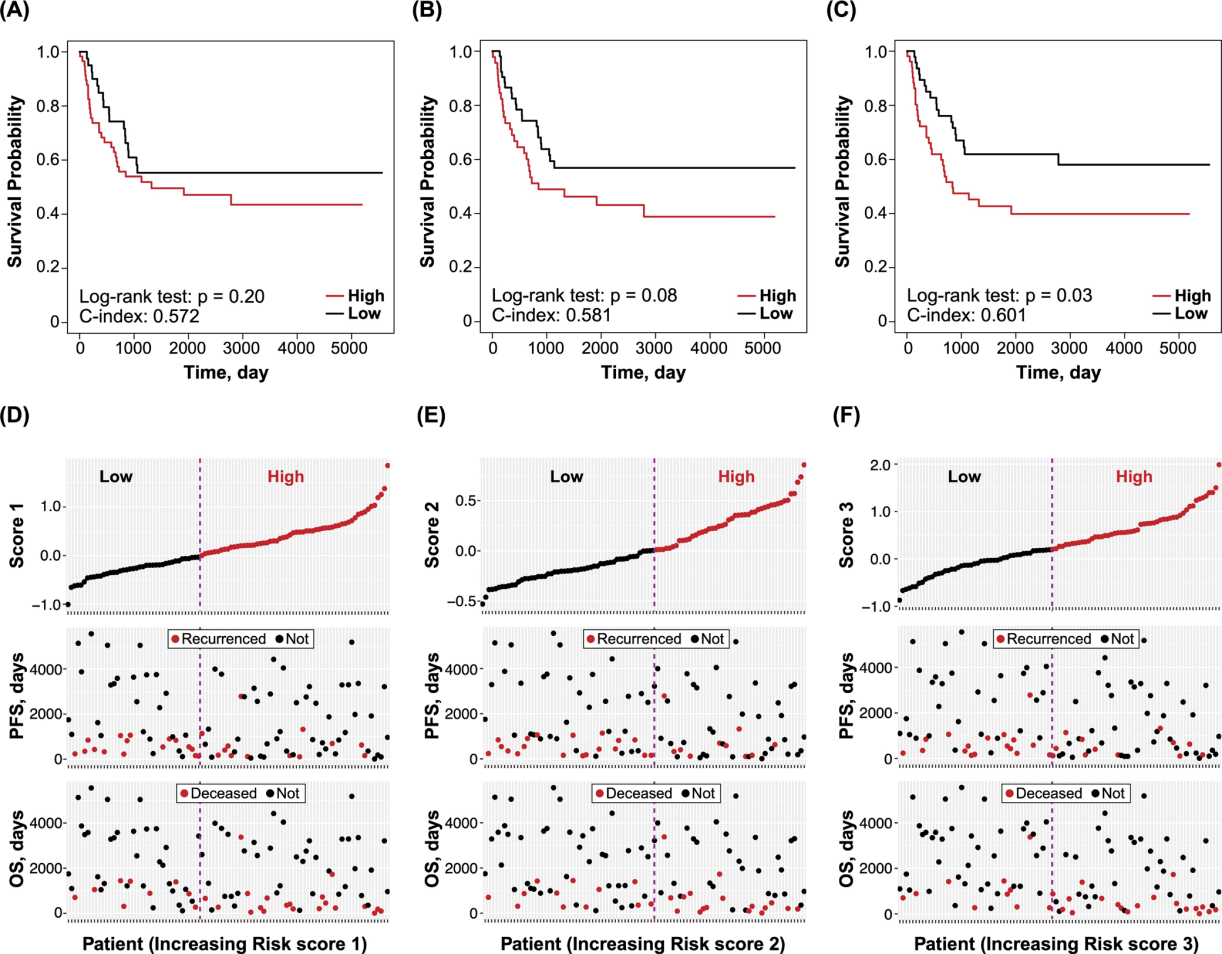
External validation of developed risk scores. (A–C) Kaplan–Meier curves stratified by high‐ and low‐risk groups based on each risk score in the validation data set. (D–F) PFS and OS plotted against patient ID sorted by each risk score value in the validation data set.

Finally, the developed risk scores were tested on an external validation cohort data set obtained from different hospitals. As illustrated in Figure 4A–C, the high‐risk group identified by the same threshold mentioned earlier exhibited shorter survival (PFS) across all three risk‐scoring systems; however, a significant difference was observed only for Risk Score 3. This finding was further corroborated when PFS and OS were plotted against patient IDs sorted by the risk score; the downward trend (graph declining to the right) in the scatter plot was most pronounced for Risk Score 3. Although not statistically significant, it is noteworthy that Risk Score 2 also exhibited a marginal *p* value in the log‐rank test. Therefore, the clinical significance of the developed risk factors was confirmed through the utilization of the external data set.

## 4 Discussion

Predicting long‐term prognosis is essential for advancing precision and personalized medicine, particularly in diseases like OCCC where prognosis can significantly vary even within the same clinical stages [6, 7, 8, 9]. In this study, prognostic risk scores were developed using different strategies and compared between the derivation set and an external validation data set obtained from a distinct hospital. Following variable selection and coefficient estimation using the derivation cohort data set, all three generated risk scores were confirmed to accurately reflect the prognosis of OCCC during internal validation with the derivation set. External validation using a separate data set revealed that risk scores employing ML‐based variable selection methods, specifically Risk Scores 2 and 3, outperformed conventional feature selection methods based on clinicians' intuition.

In computing Risk Score 3, factors available only postprimary surgery, such as surgical completeness and ascites cytology results, are taken into account. Consequently, it is reasonable that Risk Score 3 exhibited the highest efficacy and effectively distinguished high‐ and low‐risk groups of OCCC with statistical significance. However, the utility of this score remains valid and is primarily applicable in postoperative patient management and follow‐up. When comparing Risk Scores 1 and 2, marginal significance was observed solely in the case of Risk Score 2 during external validation, indicating the reliability of risk scores generated through ML‐based variable selection via a specific algorithm, as opposed to employing arbitrary methods.

OCCC typically affects a younger demographic compared to other types of epithelial OvCa [10]. In this population, considerations regarding fertility preservation may arise when determining surgical procedures and adjuvant chemotherapy regimens [21]. In addition, there exists prognostic heterogeneity among patients with stage I disease, which is the most common clinical stage for OCCC [6, 7, 8, 9]. Given these facts, accurately identifying the high‐risk group for early disease progression before initiating treatment holds significant clinical importance. Several prior studies, including research from our own group, utilized preoperative laboratory test results for this purpose [14, 16, 17]. However, these studies often lacked robust validation steps. The Risk Score 2 developed in this study utilizes only four variables obtained from general preoperative blood tests and demonstrated marginal significance during external validation. This suggests its potential utility in routine clinical practice.

One notable strength of this study lies in the implementation of a centralized pathological review system, made possible through collaboration with the Tokai Ovarian Tumor Study Group. This approach ensures consistent histological evaluation, addressing the challenge of histotype mixing in OCCC [22, 23, 24] and enhancing the reliability of our prognostic scores. Additionally, we acquired a completely external validation data set, and the obtained results were confirmed, thus ensuring the robustness of the developed scores, as previously mentioned. However, the retrospective nature of our study introduces potential limitations, including the presence of confounding factors such as medical comorbidities. Also, the variability between models should be noted; for example, the contribution tendency of cholesterol values to the final score was opposite when compared with Risk Score 1, 3 versus 2. This may stem from our strategy: conducting feature selection and coefficient estimation separately. In addition, it should be noted that the number of participants is relatively small compared with the statistically ideal number of observations upon developing a prediction model [25]. To minimize concerns over the fitting issue, we utilized a penalized regression model and conducted complete external validation of our developed model. Future prospective studies are planned to further validate our results and explore their applicability.

Our study introduces novel prognostic risk scores for OCCC aimed at identifying the high‐risk group for tumor relapse through systematic variable selection using penalized regression models. These findings underscore the importance and practicality of these scores in tailoring treatment strategies for OCCC, a condition characterized by diverse prognoses. These risk scores hold promise for improving clinical decision‐making and advancing the objectives of precision and personalized medicine in gynecologic oncology.

## Author Contributions

**Shohei Iyoshi:** conceptualization (equal), funding acquisition (equal), investigation (equal), writing – original draft (equal). **Ryo Emoto:** formal analysis (equal), investigation (equal). **Nobuhisa Yoshikawa:** conceptualization (equal), investigation (equal), project administration (equal). **Kosuke Yoshida:** data curation (equal). **Masato Yoshihara:** data curation (equal). **Satoshi Tamauchi:** data curation (equal). **Akira Yokoi:** data curation (equal). **Yusuke Shimizu:** data curation (equal). **Yoshiki Ikeda:** data curation (equal). **Kaoru Niimi:** data curation (equal). **Michiyasu Kawai:** data curation (equal). **Shigeyuki Matsui:** formal analysis (equal). **Hiroaki Kajiyama:** project administration (equal).

## Ethics Statement

The study was conducted according to the guidelines of the Declaration of Helsinki, and approved by the Institutional Review Board of Nagoya University Hospital (2006‐0357). Patient consent was waived because data collection was retrospective.

## Conflicts of Interest

The authors declare no conflicts of interest.

## Supporting information


**Figure S1:** The results of 10‐fold cross validation (CV) used to pick the variables for the Risk Score 3. ALB, albumin; ALT: alanine aminotransferase, AST: Aspartate aminotransferase; BUN, blood urine nitrogen; Chol, total cholesterol; CRE, creatinine; HB, hemoglobin; Hct, hematocrit; LDH, lactate dehydrogenase; MPV, mean platelet volume; Neu, neutrophil count; PDW, platelet distribution width; PT, prothrombin time; RBC, red blood cell; UP, urinary protein; USG, urine‐specific gravity; WBC, white blood cell. Variables selected more than five times were incorporated into the Risk Score 3 calculation formula.

**Table S1:** Comparison of baseline characteristic of patients in high‐ and low‐risk groups determined by each risk score.

**Table S2:** Summary statistics of each variable in the derivation and validation sets.

## Data Availability

The data that support the findings of this study are available upon request from the corresponding author.
