# Supplementary figures and images for: Development and Validation of Predictive Risk Scores for Ovarian Clear Cell Carcinoma: A Penalized Regression Model
Source: Cancer Med. 2025 Aug 7;14(15):e71118. doi: 10.1002/cam4.71118 (PMC12332182; doi:10.1002/cam4.71118)

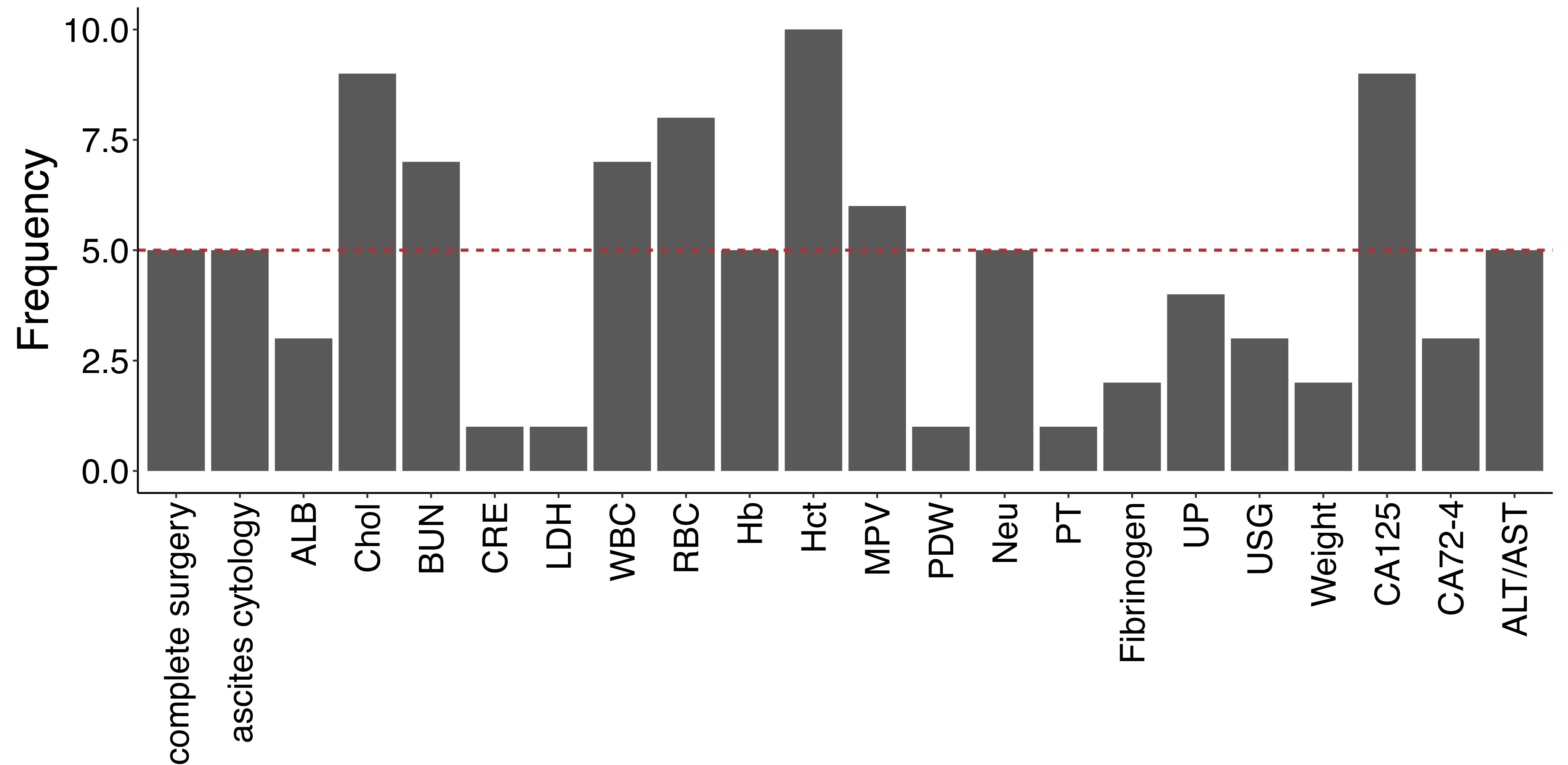

Supplement: Supplementary file 1 — Figure S1: The results of 10‐fold cross validation (CV) used to pick the variables for the Risk Score 3. ALB, albumin; ALT: alanine aminotransferase, AST: Aspartate aminotransferase; BUN, blood urine nitrogen; Chol, total cholesterol; CRE, creatinine; HB, hemoglobin; Hct, hematocrit; LDH, lactate dehydrogenase; MPV, mean platelet volume; Neu, neutrophil count; PDW, platelet distribution width; PT, prothrombin time; RBC, red blood cell; UP, urinary protein; USG, urine‐specific gravity; WBC, white blood cell. Variables selected more than five times were incorporated into the Risk Score 3 calculation formula. [file CAM4-14-e71118-s001.jpg]
