# Supplementary material for: Development and Validation of Predictive Risk Scores for Ovarian Clear Cell Carcinoma: A Penalized Regression Model
Source: Cancer Med. 2025 Aug 7;14(15):e71118. doi: 10.1002/cam4.71118 (PMC12332182; doi:10.1002/cam4.71118)
Supplement: Supplementary file 2 — Table S1: Comparison of baseline characteristic of patients in high‐ and low‐risk groups determined by each risk score. [file CAM4-14-e71118-s003.docx]

**Table S1**. Comparison of Baseline characteristic of patients in high and low risk groups determined by each risk score

|  |  |  |  | **Derivation cohort** | |  | **Validation cohort** | |  |  |
| --- | --- | --- | --- | --- | --- | --- | --- | --- | --- | --- |
|  |  |  |  | High risk | Low risk |  | High risk | Low risk |  | p-value |
| **Risk score 1** |  | n |  | 55 | 52 |  | 58 | 41 |  |  |
|  |  | Age (mean (SD)) |  | 52.24 (9.94) | 55.55 (9.47) |  | 53.97 (12.52) | 56.68 (9.87) |  | 0.185 |
|  |  | FIGO stage (%) |  |  |  |  |  |  |  | 0.013 |
|  |  | I |  | 34 (61.8) | 42 (80.8) |  | 29 (50.0) | 34 (82.9) |  |  |
|  |  | II |  | 7 (12.7) | 1 ( 1.9) |  | 8 (13.8) | 3 ( 7.3) |  |  |
|  |  | III |  | 12 (21.8) | 9 (17.3) |  | 17 (29.3) | 3 ( 7.3) |  |  |
|  |  | IV |  | 2 ( 3.6) | 0 ( 0.0) |  | 4 ( 6.9) | 1 ( 2.4) |  |  |
|  |  | Incomplete surgery (%) |  | 20 (36.4) | 19 (36.5) |  | 41 (70.7) | 14 (34.1) |  | <0.001 |
|  |  | Ascites accumulation (%) |  | 46 (83.6) | 30 (57.7) |  | 47 (81.0) | 24 (58.5) |  | 0.002 |
|  |  | Positive ascites cytology (%) |  | 32 (58.2) | 26 (50.0) |  | 32 (55.2) | 18 (43.9) |  | 0.527 |
|  |  | CA125 (median [IQR]) |  | 98.00 [52.75, 271.70] | 22.40 [12.90, 50.50] |  | 202.00 [88.20, 516.60] | 29.00 [16.00, 95.70] |  | <0.001 |
|  |  | CA19-9 (median [IQR]) |  | 33.00 [15.00, 93.00] | 21.00 [8.00, 40.00] |  | 44.25 [20.62, 154.98] | 19.90 [7.30, 61.55] |  | 0.005 |
|  |  |  |  |  |  |  |  |  |  |  |
| **Risk score 2** |  | n |  | 54 | 53 |  | 46 | 53 |  |  |
|  |  | Age (mean (SD)) |  | 52.61 (10.00) | 55.11 (9.55) |  | 54.74 (13.22) | 55.40 (9.93) |  | 0.524 |
|  |  | FIGO stage (%) |  |  |  |  |  |  |  | <0.001 |
|  |  | I |  | 33 (61.1) | 43 (81.1) |  | 19 (41.3) | 44 (83.0) |  |  |
|  |  | II |  | 7 (13.0) | 1 ( 1.9) |  | 7 (15.2) | 4 ( 7.5) |  |  |
|  |  | III |  | 12 (22.2) | 9 (17.0) |  | 15 (32.6) | 5 ( 9.4) |  |  |
|  |  | IV |  | 2 ( 3.7) | 0 ( 0.0) |  | 5 (10.9) | 0 ( 0.0) |  |  |
|  |  | Incomplete surgery (%) |  | 23 (42.6) | 16 (30.2) |  | 35 (76.1) | 20 (37.7) |  | <0.001 |
|  |  | Ascites accumulation (%) |  | 46 (85.2) | 30 (56.6) |  | 38 (82.6) | 33 (62.3) |  | 0.001 |
|  |  | Positive ascites cytology (%) |  | 34 (63.0) | 24 (45.3) |  | 26 (56.5) | 24 (45.3) |  | 0.181 |
|  |  | CA125 (median [IQR]) |  | 94.80 [52.52, 244.95] | 23.75 [13.43, 56.80] |  | 284.00 [130.00, 637.00] | 32.90 [17.00, 95.70] |  | <0.001 |
|  |  | CA19-9 (median [IQR]) |  | 36.50 [16.00, 91.75] | 18.50 [6.25, 40.50] |  | 44.70 [23.10, 150.00] | 28.00 [7.55, 66.85] |  | 0.001 |
|  |  |  |  |  |  |  |  |  |  |  |
| **Risk score 3** |  | n |  | 52 | 55 |  | 52 | 47 |  |  |
|  |  | Age (mean (SD)) |  | 52.49 (9.93) | 55.14 (9.61) |  | 55.02 (12.93) | 55.17 (9.86) |  | 0.504 |
|  |  | FIGO stage (%) |  |  |  |  |  |  |  | 0.01 |
|  |  | I |  | 31 (59.6) | 45 (81.8) |  | 25 (48.1) | 38 (80.9) |  |  |
|  |  | II |  | 6 (11.5) | 2 ( 3.6) |  | 8 (15.4) | 3 ( 6.4) |  |  |
|  |  | III |  | 13 (25.0) | 8 (14.5) |  | 15 (28.8) | 5 (10.6) |  |  |
|  |  | IV |  | 2 ( 3.8) | 0 ( 0.0) |  | 4 ( 7.7) | 1 ( 2.1) |  |  |
|  |  | Incomplete surgery (%) |  | 25 (48.1) | 14 (25.5) |  | 43 (82.7) | 12 (25.5) |  | <0.001 |
|  |  | Ascites accumulation (%) |  | 42 (80.8) | 34 (61.8) |  | 43 (82.7) | 28 (59.6) |  | 0.011 |
|  |  | Positive ascites cytology (%) |  | 36 (69.2) | 22 (40.0) |  | 33 (63.5) | 17 (36.2) |  | 0.001 |
|  |  | CA125 (median [IQR]) |  | 101.60 [51.25, 297.45] | 29.75 [15.85, 59.82] |  | 203.20 [94.25, 456.30] | 31.50 [16.15, 114.45] |  | <0.001 |
|  |  | CA19-9 (median [IQR]) |  | 34.00 [15.00, 87.25] | 20.00 [7.00, 48.25] |  | 44.30 [19.88, 170.32] | 28.00 [8.10, 55.88] |  | 0.007 |
