# Supplementary material for: Development and Validation of Predictive Risk Scores for Ovarian Clear Cell Carcinoma: A Penalized Regression Model
Source: Cancer Med. 2025 Aug 7;14(15):e71118. doi: 10.1002/cam4.71118 (PMC12332182; doi:10.1002/cam4.71118)
Supplement: Supplementary file 3 — Table S2: Summary statistics of each variable in the derivation and validation sets. [file CAM4-14-e71118-s002.docx]

|  | **Derivation cohort** | **Validation cohort** | **P-value** |
| --- | --- | --- | --- |
| **category** | (n = 107) | (n = 99) |  |
| WBC (mean (SD)) | 6678.50 (2666.53) | 6956.12 (2571.82) | 0.45 |
| Neu (mean (SD)) | 4.76 (3.11) | 6.24 (4.42) | 0.082 |
| RBC (mean (SD)) | 427.85 (42.71) | 437.27 (48.36) | 0.14 |
| Hct (mean (SD)) | 37.43 (3.93) | 37.66 (4.62) | 0.694 |
| Hb (mean (SD)) | 12.06 (1.48) | 12.14 (1.83) | 0.743 |
| MPV (mean (SD)) | 9.63 (0.73) | 9.50 (0.76) | 0.218 |
| BUN (mean (SD)) | 12.70 (4.69) | 11.58 (4.00) | 0.07 |
| Chol (mean (SD)) | 199.90 (50.65) | 193.79 (36.78) | 0.349 |
| AST (mean (SD)) | 19.79 (13.64) | 19.92 (8.65) | 0.934 |
| ALT (mean (SD)) | 16.19 (14.72) | 12.92 (6.63) | 0.045 |
| CA125 (median [IQR]) | 53.25 [22.17, 171.88] | 106.20 [29.48, 271.72] | 0.059 |
| Incomplete surgery (%) | 39 (36.4) | 55 (55.6) | 0.009 |
| Positive ascites cytology (%) | 58 (54.2) | 50 (50.5) | 0.695 |

**Table S2.** Summary statistics of each variable in the derivation and validation sets

Data are presented as mean ± standard deviation or proportion (%).

Student’s t-test, Mann-Whitney U test, chi-square test, or Fisher's exact test was used as appropriate.

Abbreviations: SD, standard deviation; WBC, white blood cell; Neu, neutrophil count; RBC, red blood cell; Hct, hematocrit; Hb, hemoglobin; MPV, mean platelet volume; BUN, blood urine nitrogen; Chol, total cholesterol; AST, Aspartate aminotransferase; ALT, alanine aminotransferase; CA, cancer antigen.
